# Probability calibration-based prediction of recurrence rate in patients with diffuse large B-cell lymphoma

**DOI:** 10.1186/s13040-021-00272-9

**Published:** 2021-08-13

**Authors:** Shuanglong Fan, Zhiqiang Zhao, Yanbo Zhang, Hongmei Yu, Chuchu Zheng, Xueqian Huang, Zhenhuan Yang, Meng Xing, Qing Lu, Yanhong Luo

**Affiliations:** 1 Department of Health Statistics, School of Public Health, Shanxi Medical University, Taiyuan, China; 2 Shanxi Provincial Key Laboratory of Major Diseases Risk Assessment, Taiyuan, China; 3 Department of Hematology, Shanxi Cancer Hospital, Taiyuan, China; 4 Department of Epidemiology and Biostatistics, Michigan State University, East Lansing, USA

**Keywords:** DLBCL, Risk prediction, Probability calibration, Discrimination and calibration

## Abstract

**Background:**

Although many patients receive good prognoses with standard therapy, 30–50% of diffuse large B-cell lymphoma (DLBCL) cases may relapse after treatment. Statistical or computational intelligent models are powerful tools for assessing prognoses; however, many cannot generate accurate risk (probability) estimates. Thus, probability calibration-based versions of traditional machine learning algorithms are developed in this paper to predict the risk of relapse in patients with DLBCL.

**Methods:**

Five machine learning algorithms were assessed, namely, naïve Bayes (NB), logistic regression (LR), random forest (RF), support vector machine (SVM) and feedforward neural network (FFNN), and three methods were used to develop probability calibration-based versions of each of the above algorithms, namely, Platt scaling (Platt), isotonic regression (IsoReg) and shape-restricted polynomial regression (RPR). Performance comparisons were based on the average results of the stratified hold-out test, which was repeated 500 times. We used the AUC to evaluate the discrimination ability (i.e., classification ability) of the model and assessed the model calibration (i.e., risk prediction accuracy) using the H-L goodness-of-fit test, ECE, MCE and BS.

**Results:**

Sex, stage, IPI, KPS, GCB, CD10 and rituximab were significant factors predicting the 3-year recurrence rate of patients with DLBCL. For the 5 uncalibrated algorithms, the LR (ECE = 8.517, MCE = 20.100, BS = 0.188) and FFNN (ECE = 8.238, MCE = 20.150, BS = 0.184) models were well-calibrated. The errors of the initial risk estimate of the NB (ECE = 15.711, MCE = 34.350, BS = 0.212), RF (ECE = 12.740, MCE = 27.200, BS = 0.201) and SVM (ECE = 9.872, MCE = 23.800, BS = 0.194) models were large. With probability calibration, the biased NB, RF and SVM models were well-corrected. The calibration errors of the LR and FFNN models were not further improved regardless of the probability calibration method. Among the 3 calibration methods, RPR achieved the best calibration for both the RF and SVM models. The power of IsoReg was not obvious for the NB, RF or SVM models.

**Conclusions:**

Although these algorithms all have good classification ability, several cannot generate accurate risk estimates. Probability calibration is an effective method of improving the accuracy of these poorly calibrated algorithms. Our risk model of DLBCL demonstrates good discrimination and calibration ability and has the potential to help clinicians make optimal therapeutic decisions to achieve precision medicine.

## Background

Diffuse large B-cell lymphoma (DLBCL) remains a clinical challenge due to its heterogeneous manifestations and prognosis [1, 2]. Although durable remission can be obtained in more than 50% of cases, relapse still occurs in 30–50% of patients with standard therapy, which dramatically reduces their survival rates [3, 4]. Autologous hematopoietic stem cell transplantation (AHSCT), second-line therapy or clinical trials are recommended for these patients with poor response [5, 6]. The accurate prediction of the risk of recurrence in DLBCL patients is crucial to clinical decision-making, as it is part of a growing trend toward precision medicine [7]. If patients with high risk of recurrence can be identified as early as possible, their prognosis would be effectively improved by taking appropriate measures e.g. AHSCT. Given that many cases may have recurrences in 3 years, thus, a model that can predict the 3-year recurrence rate of DLBCL patients is urgently required.

Statistical or computational models are powerful tools for assessing patient prognosis by simultaneously considering a number of individual features, such as demographic characteristics, disease symptoms and laboratory results. Although many studies have applied statistical models for clinical predictions, many have only focused on whether an event of interest will occur and ignored the estimate of absolute risk of this event. In many scenarios, we need to recognize whether an event will occur and obtain the membership probability, which is critical for further decision-making. For example, rather than providing a vague prognosis of survival, if we are able to predict that a patient’s 3-year survival rate with a given therapy is 50.1%, we may switch regimens early and choose a more effective regimen. Accurate risk prediction is critical for achieving precision medicine, which can help clinicians make optimal therapeutic determinations. Given accurate information, appropriate therapies may be initiated sooner, thereby preventing unnecessary exposure to ineffective drugs and ultimately improving the clinical outcomes of personalized cases and extending their survival times [7–9].

Such a clinical prediction model should be characterized by correctly distinguishing patients who will have an event from those who will not (i.e., discrimination) and by accurately estimating the absolute risk of the event (i.e., calibration) [10]. Discrimination and calibration are both necessary components of the accuracy for a risk prediction model. However, in practice, a model with good classification ability may not necessarily generate precise probability estimates, such as random forest and support vector machine models. Fortunately, these biased algorithms can be corrected by probability calibration methods. Probability calibration attempts to find a mapping function that transforms the initial risk estimates into more accurate posterior probabilities. With probability calibration, it is possible to accurately estimate the risk of recurrence of DLBCL patients even for a poor-calibrated algorithm.

Many approaches have been proposed for the probability calibration problem. Among them, Platt scaling (Platt) is a popular parametric method, which is originally proposed for SVM models [11]. Platt transforms the initial prediction into accurate posterior probability by using a sigmoid function. This method performs well when the distribution of the original probabilities is sigmoid-shaped. IsoReg (isotonic regression), the monotone extension of HistBin (histogram binning), is a popular nonparametric method [12, 13]. Since the only restriction is that the calibration function is isotonic (i.e., nondecreasing), IsoReg have the ability to calibrate any classifiers. Subsequently, Jiang [14] proposed SmoIsoReg (smooth isotonic regression), which is a continuousness extension of the IsoReg. SmoIsoReg first trains an IsoReg model and selects a set of representative points based on the piecewise constant solution generated by IsoReg. Then, the calibration function is estimated by applying PCHIP [15] interpolation algorithm to fit these points. In addition, state-of-the-art approaches such as BBQ (Bayesian binning in quantiles), GUESS and RPR (shape-restricted polynomial regression) have also been proposed to calibrate predictive models. BBQ [16, 17] integrates multiple HistBin models of different bins to generate calibrated probabilities. GUESS [18] first fits the distribution of the original scores of different classes, and then uses Bayes’ theorem to compute the probability (i.e., calibrated probability) that a certain score belongs to the interested class. RPR [19] uses a polynomial function as the calibration function and can theoretically calibrate the initial predictions of any distribution as the polynomial degree increases. In this article, the popular parametric method Platt, the popular nonparametric method IsoReg, and the flexible RPR were used to calibrate the risk prediction model for accurately predicting the 3-year recurrence rate of DLBCL patients.

Overall, we will use 5 traditional machine learning algorithms to predict the 3-year recurrence rate of patients with DLBCL: naïve Bayes (NB), logistic regression (LR), random forest (RF), support vector machine (SVM) and feed-forward neural network (FFNN) models. Previous studies showed that all of these algorithms have good classification ability; however, to our knowledge, they are rarely used for risk estimation. Thus, we will explore their calibration performance using our real-world data. Moreover, three methods (i.e., Platt, IsoReg and RPR) will be applied to develop probability calibration-based versions of each of the above algorithms. We will use the Hosmer-Lemeshow (H-L) goodness-of-fit test, expected calibration error (ECE), maximum calibration error (MCE) and Brier score (BS) to comprehensively assess the accuracy of the risk prediction. We will also explore the performance of all models on different probabilistic intervals.

This research has three objectives. First, unlike other studies that only focused on the prediction of categories, we aim to generate accurate probability estimates. Second, instead of using traditional methods, we will develop probability calibration-based machine learning algorithms for risk prediction. Third, both discrimination and calibration will be considered in the performance measure.

## Methods

### Study populations and predictors

The dataset used in this study was provided by Shanxi Cancer Hospital, China. A total of 510 patients diagnosed with DLBCL between 2011 and 2017 were included in the model construction. There were 181 cases, which had experienced relapse within 3 years. We collected 15 features of each patient from their electronic medical records. Table 1 shows the names and groupings of each feature.

**Table 1:** Features and groupings of 510 patients with DLBCL

Features	Instances (N)
Age	≤ 60 (288), > 60 (222)
Sex	Male (262), Female (248)
Stage	I (50), II (179), III (87), IV (194)
IPI	Low (255), Low-intermediate (102), High-intermediate (101), High (52)
KPS	≥ 80 (419), < 80 (91)
WBC	Low (100), Normal (377), High (33)
LDH	Normal (389), High (121)
*β*_2_-MG	Normal (373), High (137)
ESR	Normal (321), High (189)
GCB	Yes (302), No (208)
CD10	Negative (339), Positive (171)
Bcl-6	Negative (87), Positive (423)
MUM-1	Negative (276), Positive (234)
Ki-67	< 50 (53), 50 ~ 80 (165), > 80 (292)
Rituximab	Not use (290), Use (220)
Relapse	No (329), Yes (181)

*IPI* international prognostic index, *KPS* Karnofsky performance status, *WBC* white blood cell, *LDH* lactate dehydrogenase, *β*_*2*_*-MG* β_2_- microglobulin, *ESR* erythrocyte sedimentation rate, *GCB* germinal center B-cell-like lymphoma; CD10, Bcl-6, MUM-1 and Ki-67 are immunohistochemical indicators; The figures in brackets represent the number of patients of this group

We employed a LR model and RF algorithm to analyze these variables. The LR model can detect possible causal relationships between variables and identify important variables related to the outcome [20]. Table 2 shows the selected variables of the LR model when the threshold is 0.1. Sex, stage, IPI, KPS, GCB, CD10 and rituximab were significant factors for recurrence in DLBCL patients within 3 years. Except for stage-II, the *P* values of other variables were all less than 0.05.

**Table 2:** Variables selected by the LR model (*P* < 0.1)

Variable	Grouping	Coefficient	OR	*P*-value
Sex	Male	Reference	Reference	Reference
	Female	−0.466	0.628	0.037
Stage	I	Reference	Reference	Reference
	II	0.744	2.105	0.161
	III	1.573	4.823	0.006
	IV	1.429	4.175	0.011
IPI	Low	Reference	Reference	Reference
	Low-intermediate	0.907	2.478	0.008
	High-intermediate	0.953	2.594	0.013
	High	1.210	3.352	0.016
KPS	≥ 80	Reference	Reference	Reference
	<80	0.734	2.084	0.014
GCB	No	Reference	Reference	Reference
	Yes	−0.792	0.453	0.041
CD10	Negative	Reference	Reference	Reference
	Positive	−1.144	0.318	< 0.001
Rituximab	Not use	Reference	Reference	Reference
	Use	−0.502	0.605	0.027

*IPI* international prognostic index, *KPS* Karnofsky performance status, *GCB* germinal center B-cell-like lymphoma, CD10 is immunohistochemical indicators

The RF algorithm can perform feature selection by analyzing the importance of variables [20, 21]. In this research, mean decrease of accuracy and mean decrease of Gini index were selected to measure the importance of variables. The former calculates the average reduction in prediction accuracy of the model in the Out of Bag (OOB) samples after a certain variable is removed. The larger the mean decrease of accuracy, the more important the variable is to the model. The Gini index, which reflects the likelihood that two samples taken at random from a data set will have different labels, is used to measure the impurity of this data. The mean decrease of Gini index calculates the average reduction of the node impurity in all decision trees after a certain variable is used as the partition attribute. The larger the value, the more important the variable is to the model.

**Fig. 1:**
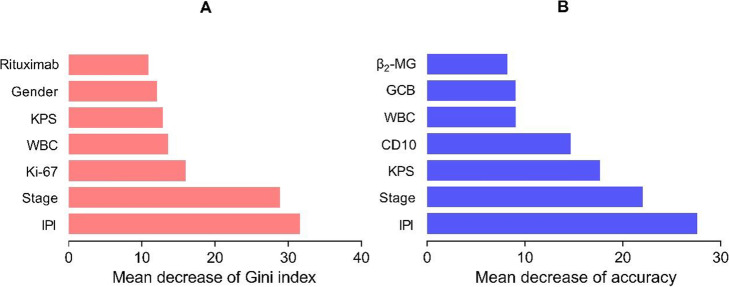
Ranking of variables importance (only showed the first 7 variables)

Figure 1 shows the ranking of variable importance. To compare with the result of the LR model, we only focused on the top 7 variables of the ranking. The union of the two rankings contained 10 variables, including 7 variables selected by the LR model, as well as WBC, Ki-67 and *β*_2_-MG. Regardless of which importance measure was used, IPI and stage were ranked in the top 2, and both rankings contained WBC and KPS.

Based on the results of these two methods, we first used the variables (sex, stage, IPI, KPS, GCB, CD10, and rituximab) selected by the LR model as the predictors of the risk model. According to these 7 variables, we pretrained the 5 machine learning algorithms with 100 times. Then, we further incorporated the WBC, Ki-67 and *β*_2_-MG variables into each algorithm to observe changes in performance. Since the predictive performances of all models were not significantly improved after included these 3 variables, we excluded them for the sake of simplicity of the model. Finally, sex, stage, IPI, KPS, GCB, CD10 and rituximab were used as the predictors to predict the 3-year recurrence rate of patients with DLBCL.

### Five machine learning algorithms

Five common machine learning algorithms that showed good classification ability in previous reports were explored, namely, the NB, LR, RF, SVM and FFNN models.

The NB classifier [22], which calculates the posterior probability that an example belongs to each member according to Bayes’ theorem, partitions the example into the member with the largest posterior probability. The LR model [23] has the “regression” term but actually belongs to a class of generalized linear models that solves classification tasks. Since it uses the logistic function as the link function, LR can generate the posterior probability that an observation belongs to a certain class.

The RF algorithm [24], which generates a series of “bootstrap” datasets of identical size as the original data based on sampling with replacement, develops a decision tree on each bootstrapped dataset. The results of all trees are voted (classification problem) or averaged (regression problem) to obtain the final prediction. In this research, the voting ratio of all decision trees was used as the probability estimate of the RF algorithm.

The SVM model [25], which is a generalization of the maximal margin classifier, attempts to find a separating hyperplane to partition samples into different classes. SVM classifies examples according to their scores *s*(***x***), which are proportional to the distance from ***x*** to the separating hyperplane. The sign of the score determines the category, and its magnitude can also be used as the measure of predictive confidence since an example far from the separating hyperplane is more likely to be classified correctly [13]. Although *s*(***x***) ∈ *R*, we can scale them into an interval between 0 and 1 by using min-max normalization.

An artificial neural network (ANN) [26] consists of a number of simple adaptive units and represents a wide parallel interconnection network. The FFNN is a common network structure in which the units in each layer are fully connected to the units in the next layer and there is no loop in the structure. In this study, we developed a 3-layer network structure, including one input layer, one hidden layer and one output layer. The hidden layer contained 1000 units, and the output layer consisted of a single unit that used the sigmoid function as the active function. Our FFNN had a large number of hidden units since the network with excess capacity has better generalization than the simple network when using back propagation and early stopping training [27–29]. Studies have showed that a multilayer feedforward network, which has a single hidden layer containing enough neurons, can approximate a continuous function with arbitrary complexity [30].

### Three probability calibration methods

We employed 3 methods (Platt, IsoReg, and RPR) to develop probability calibration-based versions of the above 5 machine learning algorithms. A total of 20 models were established in our research, including the 5 uncalibrated algorithms.

Probability calibration tries to find a mapping function that transforms the initial probability estimate or score of a classifier into more accurate prediction, i.e., find a calibration function *f* that satisfies following objective [31]:

$$ f(s)=P\ \left\{Y=1\ \right|\ S\left(\boldsymbol{x}\right)=s\Big\} $$

where *s* is the initial probability estimate or score of an example ***x***. *P* is the true probability of this example belongs to the category of interest (i.e., *Y* = 1).

Platt maps the original prediction into accurate posterior probability by using a sigmoid function [11]. The calibrated probability is generated by the following function:

$$ P\left\{Y=1|s\right\}=\frac{1}{1+\exp \left( As+B\right)} $$

The parameters A and B are estimated by using the maximum likelihood estimation (MLE) on the calibration training set ${\left\{\left({s}_i,{y}_i\right)\right\}}_{i=1}^N$. To avoid overfitting, *y*_*i*_ = (*N*_+_ + 1)/(*N*_+_ + 2) if the example belongs to the positive member; otherwise, *y*_*i*_ = 1/(*N*_−_ + 2). Constants *N*_+_ and *N*_−_ are the number of positive and negative examples in the training data, respectively.

IsoReg calibrates the initial prediction by using an isotonic (nondecreasing) function *f* that satisfies the following restriction [13]:

$$ \mathit{\operatorname{Min}}\frac{1}{N}\ \sum \limits_{i=1}^N\ {\left[\ f\left({y}_i\right)-{y}_i\right]}^2\ s.t.{f}_1\le {f}_2\le \dots \le {f}_N $$

Pair-adjacent violators (PAV) algorithm is often used to estimate the isotonic function [32]. With this algorithm, the examples are first sorted according to their initial predictions, and all positive samples have a probability of 1 and all negative samples have a probability of 0. A sequence of assigned probabilities can be obtained, i.e., *y*_*i*_ = [ *y*_1_ *y*_2_…*y*_*N*_]. Subsequently, recursively replace a pair-adjacent violator with their average of assigned probabilities, e.g., if *y*_*n*_ > *y*_*n* + 1_ (pair-adjacent violator), then update both with their average. The above replacement is executed recursively until *f*(*y*_1_) ≤ *f*(*y*_2_) ≤ … ≤ *f*(*y*_*N*_). Finally, we can obtain a stepwise constant solution over the interval of initial predictions. To predict a new example ***x***, we find the *i*-th interval in which the *s*(***x***) is located and assign *f*(*i*) as the calibrated probability for this example.

Compared to the Platt and IsoReg, RPR is a more flexible and powerful method that uses a polynomial function to calibrate a classifier [19]:

$$ f(s)={a}_0+{a}_1s+{a}_2{s}^2+\dots +{a}_k{s}^k=\sum \limits_{l=0}^k{a}_l{s}^l $$

The polynomial coefficients ***a*** are solved by the following optimization problem:

$$ \underset{\boldsymbol{a}\in {R}^{k+1}}{\operatorname{Min}}\frac{1}{N}\sum \limits_{n=1}^N\ {\left[\sum \limits_{l=0}^k{a}_l{s}_n^l-{y}_n\right]}^2 $$

1 $$ s.t.\sum \limits_{l=0}^k{a}_l\underline{s}\ge 0,\sum \limits_{l=0}^k{a}_l{\overline{s}}^l\le 1 $$

2 $$ \sum \limits_{l=1}^k{a}_l{ls}^{l-1}\ge 0,\forall s\in \left[\underline{s},\overline{s}\right] $$

3 $$ \sum \limits_{l=0}^k\left|{a}_1\right|\le \lambda $$

All calibrated probabilities are forced to fall in the interval between 0 and 1 by using the restriction (*a*). Restriction (b) derives from the differentiability of *f*(*s*), and is used to ensure the monotonicity of the calibration function. In the restriction (c), a *l*_1_-norm of coefficients is used to avoid overfitting of the polynomial.

### Model construction

The construction and evaluation of all models are completed by using the stratified hold-out test. We randomly sampled two-thirds of the observations (340) as the training data and the residual observations (170) as the testing data. To ensure the consistency of the data distribution, stratified sampling was used to partition the data. To reduce the statistical variability, the above partition and evaluation were repeated 500 times. The performance comparison was based on the average results of the 500 hold-out tests.

We first developed traditional NB, LR, RF, SVM and FFNN models for risk prediction. Threefold cross-validation was performed on the training data to determine the optimal hyperparameters of the RF, SVM and FFNN models. For the RF, the choices for the number of candidate attributes of each node partition were {2, 3}, and the number of decision trees was selected from {500, 600, 700…, 1500}. For the SVM, the kernel was selected from the linear or Gaussian kernels. The search space for the parameters C and gamma was ${\left\{{10}^i\right\}}_{i=-4}^4$. For the FFNN, the training epoch was determined by the validation sets. Subsequently, we used all training data to fit the NB and LR models and trained the RF, SVM and FFNN models with the determined hyperparameters. Finally, we assessed their performance on the testing data. To extract the predicted values of the model in the validation sets, we also performed 3-fold cross-validation on the training set for the NB and LR models, although they have no hyperparameters that need to be determined.

Then, we developed probability calibration-based versions of the above 5 algorithms. To avoid overfitting, we used the union of the predicted values on the 3 validation sets of the above 5 algorithms as the training set of the calibration function. We first employed 3-fold cross-validation on the calibration training set to determine the optimal hyperparameters of the RPR. The choices for the polynomial degree *k* were {4, 5, …, 20}, and the choices for regularization constant *λ* were ${\left\{{4}^i\right\}}_{i=0}^5$. Subsequently, we used all training data from the calibration to fit Platt, IsoReg and the RPR with the determined *k* and *λ*. Finally, we calibrated the predicted values on the testing set of the 5 algorithms by using the trained Platt, IsoReg and RPR models and then assessed their performances.

### Model evaluation

Although our purpose is to generate accurate risk estimates, classification ability is the foundation of a prediction model. When a model has a poor discrimination ability, then the accuracy of the predicted probabilities does not need to be further evaluated [10]. Thus, both discrimination ability and calibration ability of the model were considered in the performance evaluation. Discrimination is the ability to differentiate those at lower risk of an event of interest from those at higher risk. Calibration measures the similarity between predicted risk and true risk in patients in different risk strata. In our study, we used the AUC to assess the discrimination and measured the calibration by using the H-L test, ECE, MCE and BS.

The H-L test, ECE and MCE are metrics related to the calibration plot. To calculate these metrics, all examples are first sorted according to their predictions and then divided into *k* bins of similar size. In each bin, the predicted risk is the mean of the predictions of all examples in the bin and the true or observed risk is the ratio of positive members in the bin. The H-L test can measure whether the difference between the predicted risk and the true risk is caused by sampling error [33]:

$$ {C}_{H-L}=\sum \limits_{i=1}^k\sum \limits_{c=0}^1\frac{{\left({O}_i^c-{P}_i^c\right)}^2}{P_i^c} $$

${O}_i^c$ is the sum of cases with c = 0 or c = 1 in the *i*-th bin. ${P}_i^c$ is the sum of predicted probabilities with c = 0 or c = 1 in the *i*-th bin. The statistic *C*_*H* − *L*_ is then compared to a chi-square distribution with *k* − 2 degrees of freedom. The ECE and MCE calculate the average and maximum predicted errors of these bins, respectively [17]:

$$ \mathrm{ECE}=\sum \limits_{i=1}^k\mid {p}_i-{o}_i\mid /k $$

$$ \mathrm{MCE}=\max \left(|{p}_i-{o}_i|\right),i=1,2,\dots, k $$

The *p*_*i*_ and *o*_*i*_ are the predicted risk and the observed risk in the *i*-th bin, respectively. The BS is another metric to assess the calibration ability of a model:

$$ BS=\frac{1}{N}\sum \limits_{m=1}^N{\left({p}_m-{y}_m\right)}^2 $$

The *p*_*m*_ is the predicted risk of an example and the *y*_*m*_ is true label of this example. Lower ECE, MCE and BS values corresponding to a lower risk of prediction errors.

## Results

We first developed the NB, LR, RF, SVM and FFNN models and then used 3 methods (Platt, IsoReg, and RPR) to construct probability calibration-based versions of these algorithms. The performance comparison was based on the average results of the hold-out test repeated over 500 rounds. A model that obtained a H-L test value greater than 0.05 was defined as a well-calibrated model.

### Five traditional machine learning algorithms

As shown in Table 3, the AUCs of the 5 algorithms were approximately 0.75, suggesting that they achieved useful discrimination. Except for the SVM, the AUCs of the other 4 algorithms were all greater than 0.75. In terms of the AUC, the FFNN had the best classification capacity, followed by the NB model.

**Table 3:** Performance of the 5 traditional machine learning algorithms

	AUC	ECE	MCE	BS	*P*_value
NB	0.760 (0.741–0.783)	15.711 (13.557–17.914)	34.350 (29.275–39.800)	0.212 (0.199–0.228)	< 0.001(< 0.001- < 0.001)
LR	0.758 (0.733–0.779)	8.517 (7.244–10.093)	**20.100 (16.675–25.025)**	0.188 (0.180–0.196)	0.152 (0.022–0.403)
RF	0.757 (0.739–0.776)	12.740 (10.910–14.336)	27.200 (23.375–31.925)	0.201 (0.190–0.211)	< 0.001(< 0.001- < 0.001)
SVM	0.748 (0.724–0.771)	9.872 (8.317–11.777)	23.800 (19.000–28.925)	0.194 (0.185–0.204)	0.016(< 0.001–0.117)
FFNN	**0.767 (0.747–0.787)**	**8.238 (6.805–9.611)**	20.150 (16.600–24.500)	**0.184 (0.177–0.192)**	0.244 (0.075–0.518)

*NB* naïve Bayes, *LR* logistic regression, *RF* random forest, *SVM* support vector machine, *FFNN* feedforward neural network. In each cell M (*P*_25_ - *P*_75_): M is the median, *P*_25_ is the 25th percentile and *P*_75_ is the 75th percentile of 500 evaluations. The best performance in each column is in bold; The secondary best performance in each column is underlined

From the calibration, the LR and FFNN models were well calibrated. For these two algorithms, both the ECE and BS values of the FFNN were lower than those of the LR model, whereas the MCE value was slightly higher than that of the LR model. By comparison, the NB, RF and SVM models were poorly calibrated and had large errors in the probability estimate. Among them, the NB model had the lowest accuracy (ECE = 15.711, MCE = 34.350, BS = 0.212), followed by the RF model (ECE = 12.740, MCE = 27.200, BS = 0.201).

### Probability calibration-based models

Since the Platt, IsoReg and RPR methods do not change the order of the predictions of the examples, the AUCs of all calibrated models will not be discussed in this section. The results are shown in Table 4.

**Table 4:** Performance of the probability calibration-based algorithms

	ECE	MCE	BS	*P*_value
NB	15.711 (13.557–17.914)	34.350 (29.275–39.800)	0.212 (0.199–0.228)	< 0.001(< 0.001- < 0.001)
NB-Platt	9.008 (7.919–10.647)	**21.550 (17.475–25.800)**	**0.189 (0.181–0.197)**	0.179 (0.055–0.389)
NB-IsoReg	9.820 (7.740–12.190)	40.000 (23.475–57.100)	0.208 (0.195–0.227)	< 0.001(< 0.001–0.057)
NB-RPR	**8.743 (7.397–10.307)**	21.600 (17.575–25.700)	**0.189 (0.182–0.197)**	0.191 (0.051–0.431)
LR	**8.517 (7.244–10.093)**	**20.100 (16.675–25.025)**	0.188 (0.180–0.196)	0.152 (0.022–0.403)
LR-Platt	8.981 (7.478–10.485)	20.900 (17.300–25.325)	0.189 (0.182–0.196)	0.215 (0.065–0.437)
LR-IsoReg	9.140 (6.970–11.810)	31.550 (20.000–50.175)	0.204 (0.193–0.220)	0.008(< 0.001–0.348)
LR-RPR	8.744 (7.308–10.143)	20.300 (16.700–24.425)	**0.187 (0.181–0.194)**	0.255 (0.092–0.507)
RF	12.740 (10.910–14.336)	27.200 (23.375–31.925)	0.201 (0.190–0.211)	< 0.001(< 0.001- < 0.001)
RF-Platt	8.998 (7.518–10.447)	21.100 (17.500–26.700)	0.192 (0.184–0.200)	0.156 (0.030–0.435)
RF-IsoReg	9.292 (7.332–11.353)	27.850 (20.000–40.000)	0.201 (0.191–0.215)	< 0.001(< 0.001–0.131)
RF-RPR	**8.949 (7.387–10.524)**	**20.900 (17.400–26.025)**	**0.189 (0.182–0.196)**	0.194 (0.061–0.458)
SVM	9.872 (8.317–11.777)	23.800 (19.000–28.925)	0.194 (0.185–0.204)	0.016(< 0.001–0.117)
SVM-Platt	9.077 (7.702–10.895)	21.750 (17.600–27.300)	0.192 (0.184–0.201)	0.169 (0.029–0.412)
SVM-IsoReg	9.501 (7.332–12.453)	30.350 (20.000–42.200)	0.205 (0.194–0.221)	0.003(< 0.001–0.249)
SVM-RPR	**8.796 (7.362–10.439)**	**21.000 (16.775–26.550)**	**0.190 (0.183–0.199)**	0.211 (0.064–0.471)
FFNN	**8.238 (6.805–9.611)**	**20.150 (16.600–24.500)**	**0.184 (0.177–0.192)**	0.244 (0.075–0.518)
FFNN-Platt	8.991 (7.721–10.642)	20.950 (16.875–26.100)	0.186 (0.179–0.194)	0.192 (0.056–0.425)
FFNN-IsoReg	10.866 (8.603–13.347)	40.550 (27.800–57.025)	0.211 (0.196–0.230)	< 0.001(< 0.001–0.003)
FFNN-RPR	8.703 (7.393–10.361)	21.400 (17.700–26.025)	0.185 (0.178–0.193)	0.227 (0.073–0.473)

*NB* naïve Bayes, *LR* logistic regression, *RF* random forest, *SVM* support vector machine, *FFNN* feedforward neural network, *Platt* Platt scaling, *IsoReg* isotonic regression, *RPR* shape-restricted polynomial regression. “-Platt”, “-IsoReg” and “-RPR” represent performing probability calibration by using corresponding method. In each cell M(*P*_25_ - *P*_75_): M is the median, *P*_25_ is the 25th percentile and *P*_75_ is the 75th percentile of 500 evaluations. For each algorithm, the best performance in each column is in bold

Through probability calibration, the errors of the NB, RF and SVM models decreased significantly, especially for the NB model. Except for the BS value in the LR model, the calibration errors of the LR and FFNN models were not further decreased, regardless of the probability calibration method. Of the 3 calibration methods used, RPR obtained the best correction for the RF and SVM models, regardless of the ECE, MCE or BS metric. For the NB algorithm, NB-RPR had the lowest ECE, NB-Platt had the lowest MCE, and the BS values of the two models were identical. For these 3 poorly calibrated algorithms (NB, RF, and SVM), the correction effects of IsoReg were not obvious. The ECEs of the NB-IsoReg, RF-IsoReg and SVM-IsoReg models decreased compared to those of the uncalibrated models, whereas the MCEs of these models increased to different degrees. In addition, the BS value of SVM-IsoReg was also higher than that of the uncalibrated model, while the BS values of NB-IsoReg and RF-IsoReg were lower than or equal to those of the uncalibrated models.

### Improvement of the calibration

**Fig. 2:**
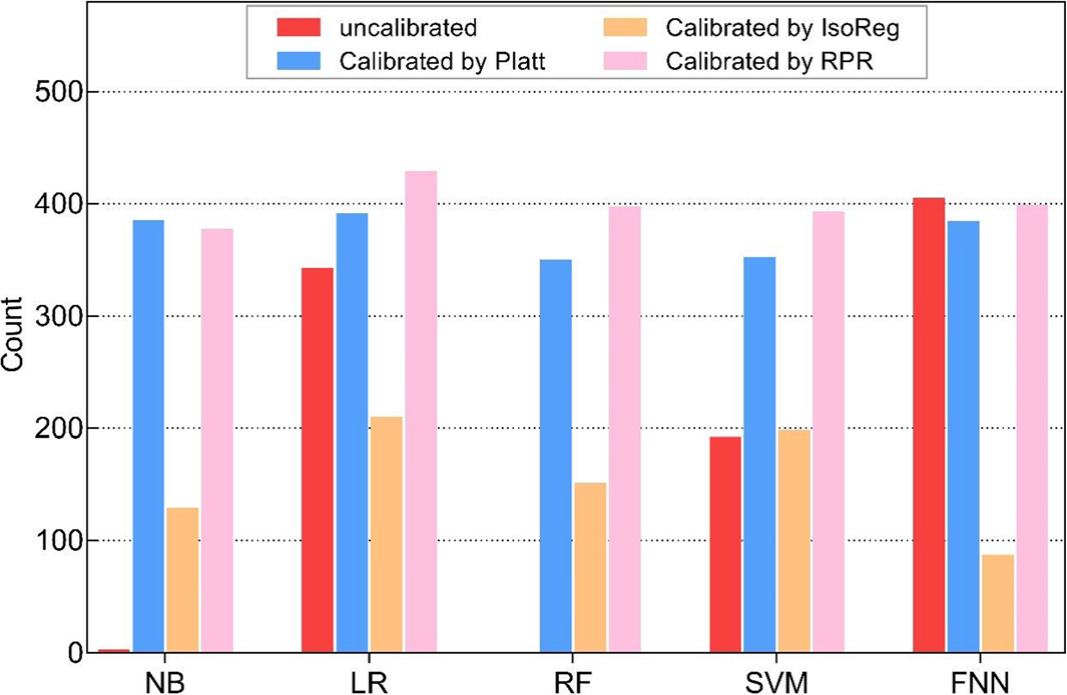
Frequency of well-calibrated models over 500 hold-out tests

We further explored improving the model calibration performance after probability calibration. In terms of the H-L test, if the result of a model was not statistically significant (*P* > 0.05), then it was defined as well-calibrated; otherwise, it was defined as poorly calibrated. Since the LR and FFNN models were well-calibrated, their calibrated models were not discussed in this section. The results are shown in Fig. 2.

For the 5 uncalibrated models, the FFNN had the highest frequency (403) of achieving a well-calibrated performance out of 500 evaluations, followed by the LR model (341). By comparison, the frequencies of the NB, RF and SVM models were 1, 0 and 190, respectively. Of these poorly calibrated algorithms (NB, RF, and SVM), the probability calibration improved their performances significantly. Compared with Platt and IsoReg, the RF-RPR and SVM-RPR models achieved the highest number of well-calibrated performances, which were 395 and 391 rounds, respectively. For the NB model, NB-Platt had the highest frequency (383), followed by NB-RPR (375).

### Distribution of probability estimates

**Fig. 3:**
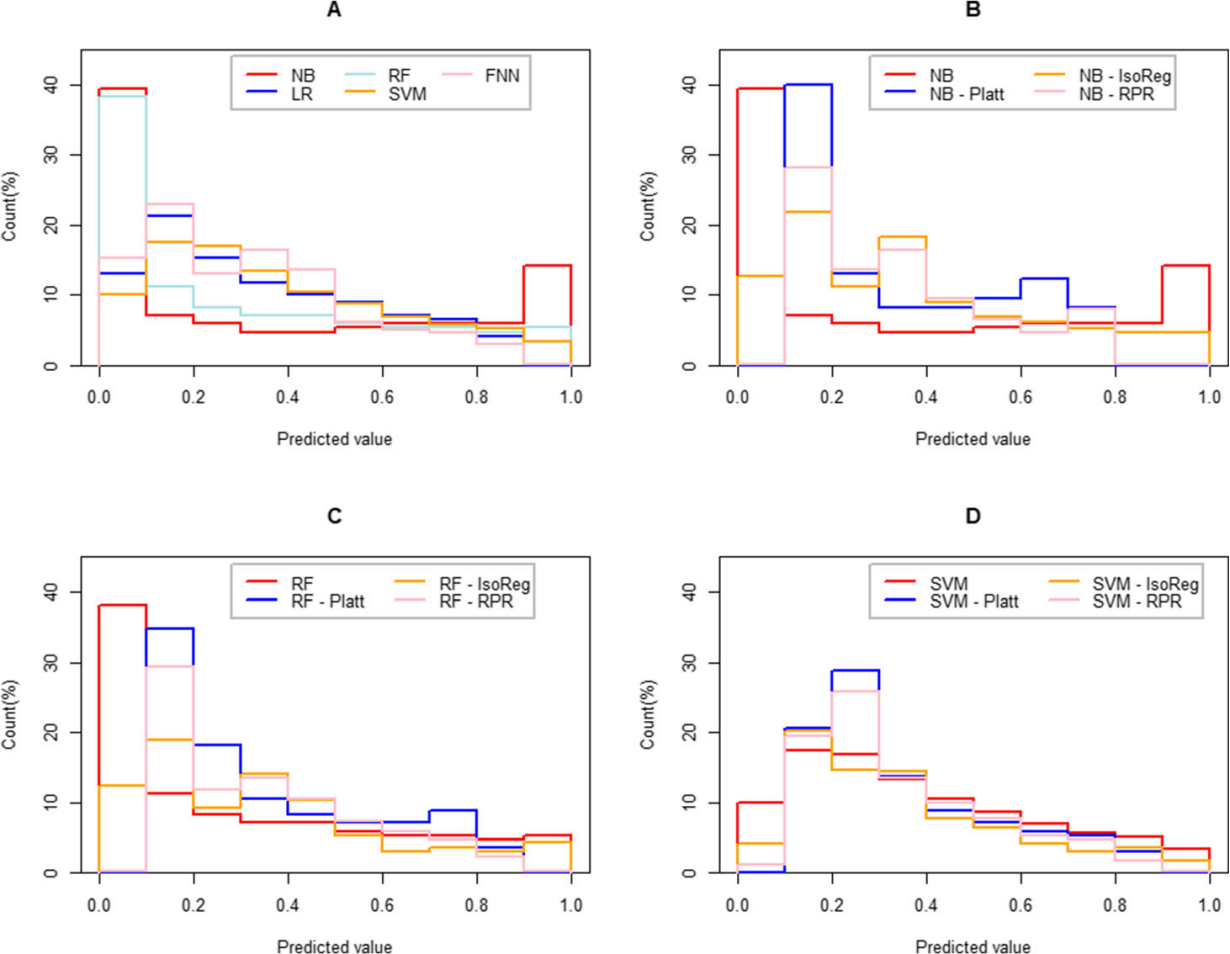
Distribution of estimated probabilities over different intervals

We finally explored the distribution of all estimated probabilities. According to the fixed cut points of 0.1, 0.2, …, 1, all examples were grouped based on their predictions. In each interval, we calculated the count of examples and expressed it using the median of 500 hold-out tests. Since the LR and FFNN models achieved good calibration, the results of their calibrated models were not discussed in this section. The results are shown in Fig. 3.

For the two well-calibrated models (LR and FFNN), the peaks clustered around the interval between 0.1 and 0.2. There was no example near the point where the predicted value was 1. Between 0.3 and 1, the numbers of examples decreased gradually as the probability increased.

For the uncalibrated NB model, the peaks were concentrated at approximately 0 and 1, and the former accounted for a larger proportion. Between 0.1 and 0.9, the count of each interval was roughly identical. For the 3 calibrated NB models, most estimated probabilities appeared in the interval between 0.1 and 0.2. For the NB-Platt and NB-RPR models, the number of examples with predicted probabilities of approximately 0 and 0.9 was 0.

For the uncalibrated RF model, the peak is approximately 0. Between 0 and 1, the count decreased gradually as the probability increased. For the 3 calibrated RF models, most estimated probabilities appeared in the interval between 0.1 and 0.2. For the RF-Platt and RF-RPR models, the number of examples with predicted probabilities of approximately 0 and 1 was 0.

For the uncalibrated SVM model, the peak at approximately 0.2. For the SVM-Platt and SVM-RPR models, most estimated probabilities appeared in the interval between 0.2 and 0.3, while the peak of the NB-IsoReg appeared in the interval between 0.1 and 0.2. For the SVM-Platt and SVM-RPR models, the number of examples with predicted probabilities of approximately 1 was 0. There were also no examples near points where the probability was 0 for the SVM-Platt model. In the interval between 0.3 and 1, the number of examples of the 4 models decreased regularly as the probability increased.

## Discussion

We developed probability calibration versions of the 5 traditional machine learning algorithms to predict the 3-year recurrence rate in patients with DLBCL and validated them in terms of both discrimination and calibration. Although the initial risk prediction of several algorithms had large errors, probability calibration improved their accuracy.

We used 7 variables, i.e., sex, stage, IPI, KPS, GCB, CD10 and rituximab, to predict the 3-year recurrence rate of patients with DLBCL. Most of these variables are associated with the clinical outcome of DLBCL. To our knowledge, the prognosis of patients is highly correlated with the tumor stage in almost all cancers. The higher the stage, the more severe the disease and the more complex the treatment; thus, a poor prognosis is likely. This fact is also true in DLBCL [34]. IPI is often used to estimate a patient’s prognosis by clinicians, and it is a recognized prognostic indictor of DLBCL [34, 35]. The IPI value is between 1 and 5, and a higher value corresponds to a greater likelihood that the patient will have a poor clinical outcome. DLBCL can be further classified into two (GCB and non-GCB) categories based on the expression of specific proteins. Significant differences in prognosis were observed between these two types, and the overall survival rate was considerably inferior in non-GCB patients [36–39]. In addition, several studies have suggested that the expression of CD10 is closely associated with patient survival and has a favorable effect on clinical outcomes [40, 41]. The application of rituximab is a breakthrough in DLBCL, and current studies have shown that rituximab improves survival in almost all DLBCL subgroups [4, 42–44]. The KPS reflects the physical condition of a patient, and a higher score corresponds to a better condition. Although few studies have focused on the correlation between KPS and DLBCL, we speculate that the performance status will affect patient treatment, such as the drug dosage, and thus indirectly affect patient prognosis.

The 5 machine learning algorithms discussed in this study are often used in classification tasks, and they all have good discrimination ability. In our research, although their discrimination performances were very similar, the differences in calibration were large. Both the LR and FFNN models were well calibrated, and their performances were not further improved after probability calibration. Their low calibration errors were more likely the result of a direct optimization for log-loss of probability [45]. By comparison, the NB, RF and SVM models were poorly calibrated, and their errors in estimated probabilities were large. The NB model only achieved good calibration once out of 500 evaluations. Studies have suggested that the predictions of the NB model are often pushed to 0 or 1 since its basic assumption (i.e., assume that each variable affects the result independently) may not be valid in reality [12, 13, 45]. In our study, the predictions of the NB model were concentrated at approximately 0 and 1, with the former accounting for a larger proportion. For the RF model, a good calibration performance was not achieved once out of 500 evaluations. To increase the difference between decision trees, the RF algorithm introduces the sample and attribute perturbations when constructing each tree. Several studies have suggested that it is difficult to get identical predictions from all trees; thus, the voting ratios of the RF are often pushed away from 0 and 1 [31, 45, 46]. However, most predictions from the RF model are concentrated at approximately 0, and the number of examples in the interval between 0.9 and 1 is not the lowest in our study. We suggest that three reasons may explain this difference. First, each decision tree of the RF model has good classification ability since our data are not complex. Despite the diversity imposed on the tree, most of them generate the same output. Second, the negative examples account for a large proportion in our study. Third, the RF model achieves high discriminative power for these negative examples. Furthermore, the SVM model pushes the outputs away from 0 and 1, which is consistent with the previous study [45]. Our study also suggests that probability calibration is necessary for the SVM algorithm since normalizing its scores is insufficient to obtain accurate probability estimates.

We selected 3 methods (Platt, IsoReg, and RPR) to develop probability calibration-based versions of 5 traditional machine learning algorithms. Platt is a popular parametric method that uses a sigmoid function to calibrate a classifier. If the distribution of the initial probability estimates is inconsistent with the assumed parametric form, however, Platt does not work well. In our study, the biased NB, RF and SVM models were well-corrected by the Platt method. If a classifier can rank examples correctly, then the mapping function from initial predictions into accurate probabilities should be nondecreasing. Based on this assumption, IsoReg uses an isotonic (i.e., nondecreasing) function to calibrate the biased prediction. Due to its simple restriction, IsoReg has become a popular nonparametric probability calibration method with good universal ability. However, the NB-IsoReg, RF-IsoReg and SVM-IsoReg models in our study were still poorly calibrated. Although the ECE values of these 3 models were all lower than those of the uncalibrated models, their MCEs were all increased. After investigation, we found that the calibration error of IsoReg for those examples with high predicted values is large. We speculate that overfitting occurred in these intervals with high predicted values since there were insufficient positive examples in our study. When the calibration set is small, the risk of IsoReg overfitting is large. Niculescu-Mizil and Caruana [45] also confirmed that IsoReg is not suitable for the case of training sizes less than 1000. By comparison, RPR is more powerful and flexible. Compared with Platt, RPR uses a polynomial function to calibrate a classifier and can theoretically correct the initial predictions of any distribution as the polynomial degree increases. Unlike IsoReg, the calibration function of RPR is continuous over the entire interval. Therefore, two examples with similar predicted values will not differ considerably after calibration. In our study, RPR achieved the best correction for the RF and SVM models in terms of ECE, MCE and BS values. For the NB model, NB-RPR was best in terms of the ECE, although its MCE was slightly higher than that of NB-Platt.

This paper focused on calibration rather than discrimination and aimed to provide accurate membership probability (i.e., the 3-year recurrence rate of patients with DLBCL). In practice, we will never know the true membership probability and we usually use the empirical probability (i.e., the proportion of positive events under a certain score or within a certain interval of score) to measure the membership probability. For a sample in which the event of interest has occurred, the true membership probability is not necessarily 100%. In fact, it may be 0.5, 0.6 or other values, just the existence of “probability” allows us to observe the occurrence of this event. In chapter 3.4, we can find in this research that there were some estimated probabilities that fell in the middle of the [0, 1] interval even if a well-calibrated model. These probabilities with moderate values such as those between 0.3 and 0.7 may be considered less confident for a classification task (assuming that the cut-off of classification is 0.5), since they are near the threshold. However, these moderate predictions would be of enormous help to clinical practice if the focus is on calibration rather than discrimination. For example, probabilities include those with moderate values can be used as the basis of patient risk stratification, e.g. patients with a predicted value of less than 0.3 can be regarded as low-risk individuals, those with a predicted value of 0.3 to 0.7 as medium-risk individuals, and those with a predicted value of more than 0.7 as high-risk individuals. Then, personalized treatments or interventions can be applied to different groups to improve the clinical outcomes of patients with distinct prognostic characteristics. Currently, estimating membership probability has received more and more attention and has critical clinical significance as the advent of precision medicine era [7]. Accurate risk estimates based on personalized characteristics can help improve individual risk counseling, stratification of patients for clinical trials, and timing of clinical intervention [7, 47]. Moreover, the exclusion of patients who are unlikely to respond to a standard treatment can minimize the exposure of patients to costly therapies that are unlikely to help them [7]. The risk model developed in our study achieved good performance on both discrimination and calibration and has the potential to improve the clinical outcomes of patients with DLBCL.

This research has limitations. First, the calibration performance can be further improved. Since the calibration function has to ensure monotonicity over the entire interval of initial predicted values, the calibrated probability of an example may not change significantly. Therefore, the calibration error will be largely influenced by those misclassified examples. We will collect more information of patients to improve the discriminative ability of the model, thus, indirectly increase the accuracy of the estimated probabilities. Second, only 5 machine learning algorithms are discussed in this study. The other algorithms and their probability-calibration-based versions can be further explored. Third, the data used in this study are provided by a certain hospital, therefore, an external validation is needed to evaluate the generalizability of the model.

## Conclusions

To accurately predict the 3-year recurrence rate of patients with DLBCL, we developed probability calibration-based versions of 5 traditional machine learning algorithms. In the current study, we could show that (i) some algorithms (i.e., NB, RF and SVM models) when predicting the 3-year recurrence rate of DLBCL patients cannot generate accurate risk estimates, although they have good discrimination capacity. The evaluation of performance via ECE, MCE and BS values showed that probability calibration improves the calibration performance of these algorithms effectively. Especially for the NB model, probability calibration reduced the ECE value from 15.711 to 8.743, the MCE value from 34.350 to 21.550, and the BS value from 0.212 to 0.189. These improvements provided by probability calibration are helpful to clinical practice, for example, DLBCL patients with high risk of recurrence would be identified more accurately (ii) Probability calibration did not further reduce the probabilistic error of the FFNN model in this research, regardless of which calibration method was used. Among the 20 models developed, the uncalibrated FFNN model performed best in terms of the ECE and BS values. This result may indicate that accurate risk estimates can be obtained directly by selecting a well-calibrated model in advance, without additional probability calibration.

## Data Availability

The dataset generated and analyzed during the current study are not publicly available due to subsequent studies have not been completed but are available from the corresponding author on reasonable request.

## Abbreviations

DLBCL: Diffuse Large B-cell Lymphoma
NB: Naïve Bayes
LR: Logistic Regression
RF: Random Forest
SVM: Support Vector Machine
FFNN: Feedforward Neural Network
Platt: Platt Scaling
IsoReg: Isotonic Regression
RPR: Shape-restricted Polynomial Regression
AUC: Area Under the Receiver-operating Characteristic Curve
H-L: Hosmer-Lemeshow
ECE: Expected Calibration Error
MCE: Maximum Calibration Error
BS: Brier Score
IPI: International Prognostic Index
KPS: Karnofsky Performance Status
WBC: White Blood Cell
LDH: Lactate Dehydrogenase
*β*_2_-MG: *β*_2_-Microglobulin
ESR: Erythrocyte Sedimentation Rate
GCB: Germinal Center B-cell-like Lymphoma
